# Passive mechanical features of single fibers from human muscle biopsies – effects of storage

**DOI:** 10.1186/1749-799X-3-22

**Published:** 2008-06-07

**Authors:** Fredrik Einarsson, Eva Runesson, Jan Fridén

**Affiliations:** 1 Department of Orthopaedics, Sahlgrenska University Hospital, Göteborg, Sweden; 2 Lundberg Laboratory for Orthopaedic Research, Göteborg, Sweden; 3 Department of Hand Surgery, Sahlgrenska University Hospital, Göteborg, Sweden

## Abstract

**Background:**

The purpose of this study was to investigate the effect of storage of human muscle biopsies on passive mechanical properties.

**Methods:**

Stress-strain analysis accompanied by laser diffraction assisted sarcomere length measurement was performed on single muscle fibres from fresh samples and compared with single fibres from stored samples (-20°C, 4 weeks) with the same origin as the corresponding fresh sample. Basic morphological analysis, including cross sectional area (CSA) measurement, fibre diameter measurement, fibre occupancy calculation and overall morphology evaluation was done.

**Results:**

Statistical analysis of tangent values in stress-strain curves, corresponding to the elastic modulus of single muscle fibres, did not differ when comparing fresh and stored samples from the same type of muscle. Regardless of the preparation procedure, no significant differences were found, neither in fibre diameter nor the relation between muscle fibres and extra-cellular matrix measured under light microscopy.

**Conclusion:**

We conclude that muscle fibre structure and mechanics are relatively insensitive to the storage procedures used and that the different preparations are interchangeable without affecting passive mechanical properties. This provides a mobility of the method when harvesting muscle biopsies away from the laboratory.

## Background

Experiments that may be considered as the foundation for changing clinical practice must rely on data and data analyses without obscuring methodological issues. Analysis of mechanical properties of human muscle tissue experiments are typically performed using fresh tissue. For practical reasons biopsies are commonly stored for subsequent analysis and therefore any factors related to storage per se affecting mechanical properties and morphology need to be addressed.

In a current laboratory set-up, we have chosen to test passive mechanical features as part of characterisation of muscles. We use stress-strain measurements of both single fibres and bundles accompanied by measurements of sarcomere length by means of laser diffraction technique as described by Yea et al. [1]. Reports of effects of storage of human muscle biopsies are scarce.

Frontera and Larsson [2] investigated human specimens, especially regarding possible variations in test results comparing three techniques for fibre preparation and storage. Their interpretative conclusion was that chemical skinning and sucrose incubation preserve the properties of single muscle fibres better than freeze-drying and that sucrose incubation may allow longer storage of fibres.

To evaluate whether storage has any effect on passive mechanical properties tests comparing fresh and stored human muscle tissue were performed. These analyses were accompanied by analyses of morphological features comparing fresh and stored biopsies. Our hypothesis was that there is no difference in passive mechanical properties between samples from the two preparations.

## Methods

### Ethics

This study was approved by the Human Ethical committee at Göteborg University (approval number S166-1). All patients gave their informed consent.

### Biopsy procedure

Open surgical biopsies were obtained from human forearm muscles of five healthy patients (age 24–68 years) undergoing surgery of the forearm (fracture surgery, plate removal and tendon transfer surgery. The surgeon exposed the muscle of interest and the parallel orientation of the muscle fibres was defined by inspection. A small part (approximately 15 × 5 × 5 mm) of the muscle was freed by alternating sharp and blunt dissection taking care not to mechanically damage the central part of the biopsy. The biopsies were then carefully divided into smaller pieces by scissors in parallel with the fibre orientation and put in a test tube with relaxing solution (cf. below).

### Muscle preparation

Samples were treated in two different ways. One part, defined as fresh (F), was taken from the relaxing solution (see below), embedded in OCT ("Optimal Cutting Temperature", a special low-temperature embedding medium for cryosectioning techniques. OCT; Miles Laboratories, Naperville, Il, USA) and frozen in isopentane (pre-cooled in liquid nitrogen).

The other part was stored in a storage solution, stored (T) in freezer at -20°C. After storage for 4 weeks the biopsies were washed in relaxing solution and then treated as described above.

### Solutions

Relaxing (or working) solution contained 7.5 mM EGTA ("Ethylene Glycol Tetraacetic Acid", a chelating agent with a high affinity for calcium and therefore useful for making buffer solutions that resemble the intracellular environment), 170 mM KPr, 2 mM MgAcetat, 5 mM Imidazole, 10 mM phosphocreatin, 4 mM Na_2 _ATP, 17 μg/ml leupeptin, 4 μg/ml E64 (E 64 is an inhibitor of the lysosomal proteinase Cathepsin B i.e., inhibitor of protein breakdown). Storage solution included the same constituents as the relaxing solution with an addition of NaN3 (to a concentration of 1 mM) and glycerol (to a concentration of 50%). This was obtained by adding 1 ml 0.5 M NaN_3_/500 ml storage solution and 250 glycerol/500 ml solution to the relaxing solution.

### Mechanical properties

The biopsy and storage procedures were identical to that for the morphology part of this study. Stored (frozen) preparations were gently defrosted on ice-bed in relaxing solution. Single fibres were dissected under microscope (Leica MZ8, Heerbrugg, Switzerland) with epi-illumination (model DCR II, Fostec, Auburn, NY) using forceps (P-00019, S&T, Neuhausen, Switzerland) and scissors. The chosen fibre was then transferred to a glass-bottomed chamber containing relaxing solution, specially designed to fit to our microscope and laser set-up. The whole set-up was placed on a vibration isolation table (Newport Instruments, Irvine, CA, USA). The fibre was then mounted to titan-thread lever arms by 10-0 monofilament sutures under microscope (Leica model MZ95, Heerbrugg, Switzerland) while still in the relaxing solution. The lever arms were connected to a force transducer (Model 405A-10 V/gram, Aurora Scientific Inc, Ontario, Canada) and a manually regulated digital micromanipulator (Mitutoyo 0–1", Tokyo, Japan) respectively.

Fibre length (knot to knot) was measured indirectly on a video monitor (Sony Trinitron Color Video Monitor, PVM-14M2 MDE, Tokyo, Japan) by magnification via a camera (Ikegami CCD Color Camera Model ICD-810P, Tokyo, Japan) attached to the microscope. Fibre diameter was measured in the same way and fibre area was calculated assuming cylindrical shape. A laser beam from a HeNe-laser (Melles Griot Model U-1507, Carlsbad, CA, USA) was then directed through the chamber hitting the mounted fibre at a right angel and creating a diffraction pattern. Sarcomere length (SL) was calculated by measurement of distance between light peak maximum as described by Yeah [1].

To determine distance between peaks of light interference a digital calliper was used. Two observations of 0 ^th ^– 1^st^, 1 ^st ^– 1 ^st ^and 0^th ^– 2^nd ^diffraction order peak intensities were made after each stretch [1].

Initial sarcomere length was defined as SL with the fibre mounted and "uncoiled" but not stretched. Tension as response to stretch was registered on a voltmeter (Amprobe AM-15, Everett, WA, USA). The fibre was then stretched in a continuous protocol recording tension values after stress relaxation of 1 minute. The stretch steps were 250 μm up to a total stretch of 4 mm and in steps of 500 μm thereafter. Stretch was discontinued at a total stretch of 8 mm or at fibre rupture. Slope of stress-strain curve was determined for each sample by defining the linear portion of the curve in the range of SL between 1.7 and 4.8 μm. Stress-strain curves are presented with stress values, based on tension at 1 minute of stress relaxation, corrected for area change during stretch assuming linear deformation of a cylinder with a constant volume. Change in sarcomere length (SL) is expressed as relative SL. The initial SL was set to 1 (unit).

### Morphology

The OCT-embedded muscle biopsies were cut in a cryostat (Microm HM 500, Walldorf, Germany) in 10 μm thick sections and put on microscope slides and stained with Haematoxylin & Eosin (HE). Each slide was inspected by two independent and trained observers under light microscope (Nikon Eclipse E 600) to which a video camera (Sony Power HAD Video cam) was attached. Muscle cross sections were measured for single fibre diameter according to Dubowitz [3] using software for PC (Easy Image measure module 2000, Bergström Instrument AB, Stockholm, Sweden). Areas in the section were chosen with emphasis on finding polygonal or circular shape of the cut fibres and avoiding areas with semicircular or longitudinal cuts. At least 150 fibres were measured on each slide. Measured cells were counted. Overall morphology was based on homogeneity of cells, presence of inflammatory cells, and position and density/number of nuclei. Atypical findings were recorded. Fibre occupancy (FOC) was calculated as a quote of fibre area (FA) per total measured area including extra-cellular matrix (ECM).

### Statistics

Data regarding fibre diameter are presented for one of the observers (FE). Data from the other observer (ER) were used to calculate inter-observer error. The diameter of muscle fibres specific to each slide is presented with number of fibres (n), mean, SEM, and FOC. Two-sided Student's t-test for paired observations was used to detect differences in fibre size mean between the different preparations of the same biopsy. Mann-Whitney U-test was used to test for difference in mean FOC.

A probability of less than 0.05 at statistical analysis of the observed outcome was considered significant.

The elastic modulus was determined as tangent of a linear portion of the stress-strain curve located within a physiological range of the sarcomere length (up to 2.5 times initial SL). Data are presented for fresh and stored biopsies.

## Results

### Mechanical property comparisons (fresh and stored)

**Figure 1:**
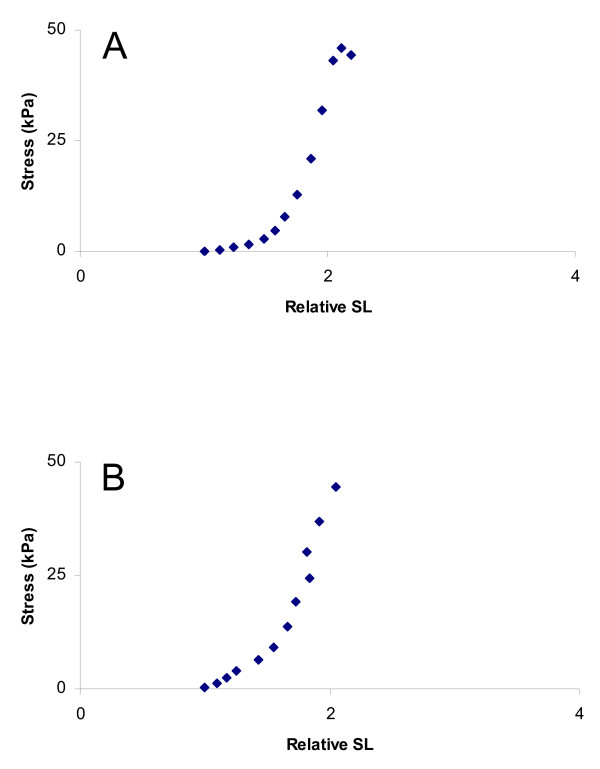
**Representative stress-strain curves**. (A) fresh and (B) stored samples from the same muscle.

Comparisons of stress-strain curves demonstrated a substantial variability between patients and muscles, but essentially identical responses between the different treatments of the biopsy samples (Fig 1). The predominant shapes of the stress-strain graphs were exponential or sigmoidal. Mean ratio for tangent modulus between stored and fresh samples was 1.12 ± 0.05 with a variation coefficient (CV) of 12%.

### Structural property comparisons

**Figure 2:**
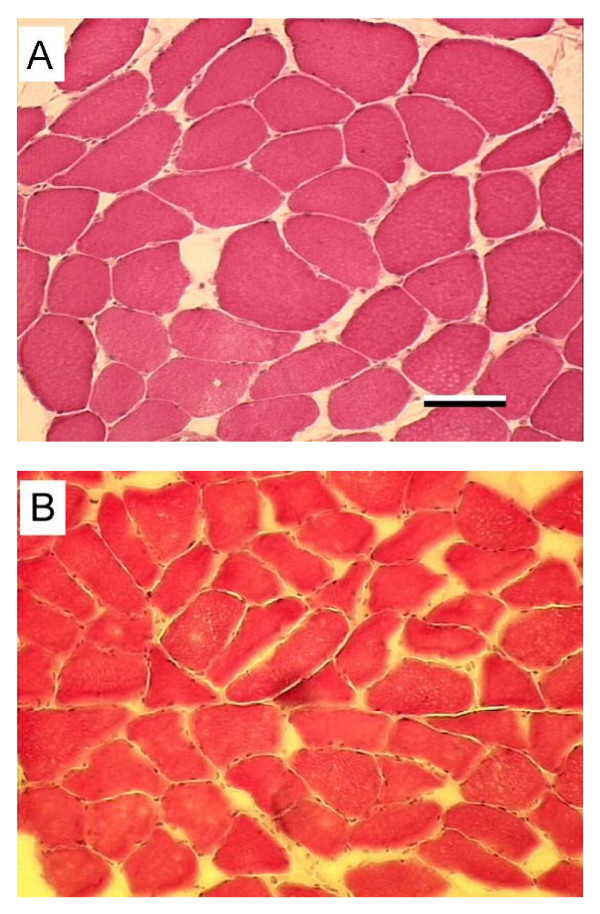
**Representative Hematoxylin-Eosin stained cryosections**. Two different treatment protocols; (A) fresh and (B) stored. Both sections are from the same muscle. Magnification bar = 100 μm.

**Figure 3:**
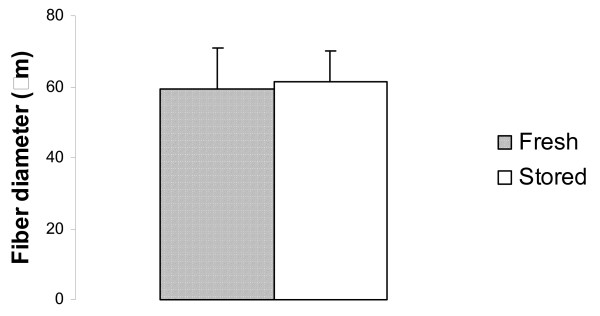
**Muscle fibre diameter for fresh and stored samples**. Mean and + SEM.

All slides used for measurements demonstrated tightly packed and usually polygonally shaped muscle fibres with normal staining characteristics (Fig. 2). The muscle fibres were organized into well-defined fascicles. Extra cellular space was sparse. A total of 1459 cells were counted (802 fresh and 657 stored). There was no significant difference in fibre diameter between skinned and stored samples. (Fig 3, Table 1 and 2). Neither were there any significant differences of FOC (%) between fresh and stored samples (94.5 ± 0.8 vs. 91.4 ± 2.7).

**Table 1:** Characteristics of individuals from which samples were analysed

Subject	Gender	Age	Muscle studied
1	F	69	FPB
2	M	56	EPL
3	M	23	Deltoid
4	M	24	ECRL
5	M	24	BR

FPB = Flexor Pollicis Brevis; EPL = Extensor Pollicis Longus, ECRL = Extensor Carpi Radialis Longus; BR = Brachioradialis

**Table 2:** Number of fibres, mean fibre diameter and SEM of the samples analysed morphologically for fresh and stored preparations.

	Fresh	Stored	TOT
N	802	657	1459
Mean	59.3	61.4	60.4
SEM	5.2	4.2	3.4

## Discussion

This study demonstrated that muscle fibres respond identically regardless of whether the biopsies are tested fresh or after storage as evidenced by roughly identical morphological and mechanical features. This observation is in line with previous observations [4] and the insensitivity to storage up to 4 weeks enable consecutive tests of several samples without obscuring interpretations due to factors related to storage.

Also studies comparing chemical skinning and storage at -20°C freeze-drying and -80°C storage found the resting tension of single fibres to be higher and maximum and specific tension to be lower after freeze drying but no find differences in cross sectional area of muscle fibres [2].

Characterization of muscle tissue is done in vivo or in vitro. Dealing with muscle biopsies both active and passive testing of mechanical properties can be performed.

It is reasonable to assume that changes in mechanical properties, in the experimental situation, might be time-dependent and related to access to energy substrate and oxygen, temperature change of the relaxing solution and presence of enzyme inhibitors. Experimentation in our set-up lasts from one up to four hours with the biopsy kept in relaxing solution on ice. This duration of experiments may cause subtotal blocking of enzymatic activity and the consumption of oxygen is likely to cause a gradual degradation of protein structure. Preparation procedure is evidently not a factor in the potential time-related deterioration under the current experimental situation. The variability observed in this study between muscles and individuals is not discussed in the present study. Furthermore, it is unknown whether damaged or diseased muscles would respond differently to storage. The present study did not investigate storage at different temperatures than -20°C or longer duration of storage than 4 weeks.

The presented data suggest that results from experiments with samples, that have been stored can be interpreted as if the sample would have been fresh. It is evident that results in terms of morphological features and passive mechanical properties of human striated skeletal muscle obtained from stored preparations correspond to those of experiments made with fresh samples and that data from either procedure reliably reflect properties of the muscle-tendon complex in vivo.

## Conclusion

In conclusion it can be stated that muscle fibre structure and mechanics are relatively insensitive to the storage procedures used and different preparations can be used interchangeable without affecting passive mechanical properties. This information provides mobility of the method when harvesting muscle biopsies in field studies.

## Competing interests

The authors declare that they have no competing interests.

## Authors' contributions

FE has participated in all parts of this manuscript including design of the study, sampling of muscle specimen, preparation of and assessment of muscle specimen, drafted the manuscript and approved of the final manuscript.

ER has participated in all parts of the manuscript with design of the study, preparation and mechanically testing and morphological investigation of the muscle specimen, performed the statistical analysis, drafted and revised the manuscript.

JF has been involved drafting the manuscript and revising it for critically for important intellectual content and giving final approval of the version to be published.
